# High throughput profiling of tocochromanols in leaves and seeds of Arabidopsis and Maize

**DOI:** 10.1186/s13007-020-00671-9

**Published:** 2020-09-17

**Authors:** Yan Bao, Maria Magallenes-Lundback, Nicholas Deason, Dean DellaPenna

**Affiliations:** Department of Biochemistry and Molecular Biology, Michigan State University, East Lansing, MI 28824 USA

**Keywords:** Vitamin E, Tocopherol, Stress, Arabidopsis, Maize, Leaf, Seed

## Abstract

**Background:**

Tocochromanols are a group of lipid-soluble antioxidants produced by all plants and include the tocopherols, which are ubiquitous in the plant kingdom, and the biosynthetically-related compounds tocotrienols, which along with tocopherols commonly occur in seed of monocots. Most tocochromanols have some level of vitamin E activity, with α-tocopherol being the highest, and as such are essential nutrients in the human diet. Tocochromanols are particularly abundant in seeds and are critical for maintaining seed longevity and proper germination and as seed oils are a major component of the human diet, they are an important source of dietary vitamin E. In vegetative tissues, tocochromanols are important components in plant responses to stressful environments and can accumulate to high levels in response to various stresses including high light, heat, and dark.

**Results:**

We report a robust, high throughput extraction and HPLC analysis method to quantify the levels of tocopherols and tocotrienols in leaves and seeds of plants, using Arabidopsis and maize tissues as examples.

**Conclusion:**

The described method provides a rapid, high-throughput, cost-effective approach to quantifying the composition and content of tocopherols, and if needed simultaneously tocotrienols, in vegetative tissues and seeds. Optimized extraction methods are described for the two tissue types and have been used to study tocochromanol (vitamin E) natural variation in seed of large Arabidopsis and maize diversity panels, to assess gene function in T-DNA and Mu-tagged populations of Arabidopsis and maize, respectfully, and study the impact of environmental stresses, including high light stress, heat stress, and dark on tocopherols content and composition of vegetative tissue.

## Background

Tocochromanols, which include tocopherols and tocotrienols, are a class of lipid-soluble, amphipathic compounds bearing a chromanol ring and polyprenyl side chain. The side chain of tocopherols is fully saturated and derived from phytyl diphosphate (phytyl-DP) while that of tocotrienols is derived from geranylgeranyl diphosphate (GGDP) and contains three additional double bonds. Tocopherols are ubiquitous in plants while tocotrienols are more restricted in occurrence and are primarily produced, along with tocopherols, by monocots. Four isoforms of tocopherols and tocotrienols (α, β, δ and γ) occur and differ in their degree and location of methylations on the chromanol ring head [1]. Most tocochromanols have vitamin E activity with α-tocopherol being the most active in this regard. Severe vitamin E deficiency in humans is caused by mutations disrupting the dietary uptake system for tocochromanols and results in severe neurological problems including ataxia, peripheral neuropathy and myopathy [2]. Because plant seeds accumulate high levels of tocochromanols and oils derived from plant seed have correspondingly high levels of tocochromanols, plant oils are a major source of vitamin E in the human diet, despite the fact that α-tocopherol is often not the major tocochromanol accumulated by plant seeds.

**Fig. 1:**
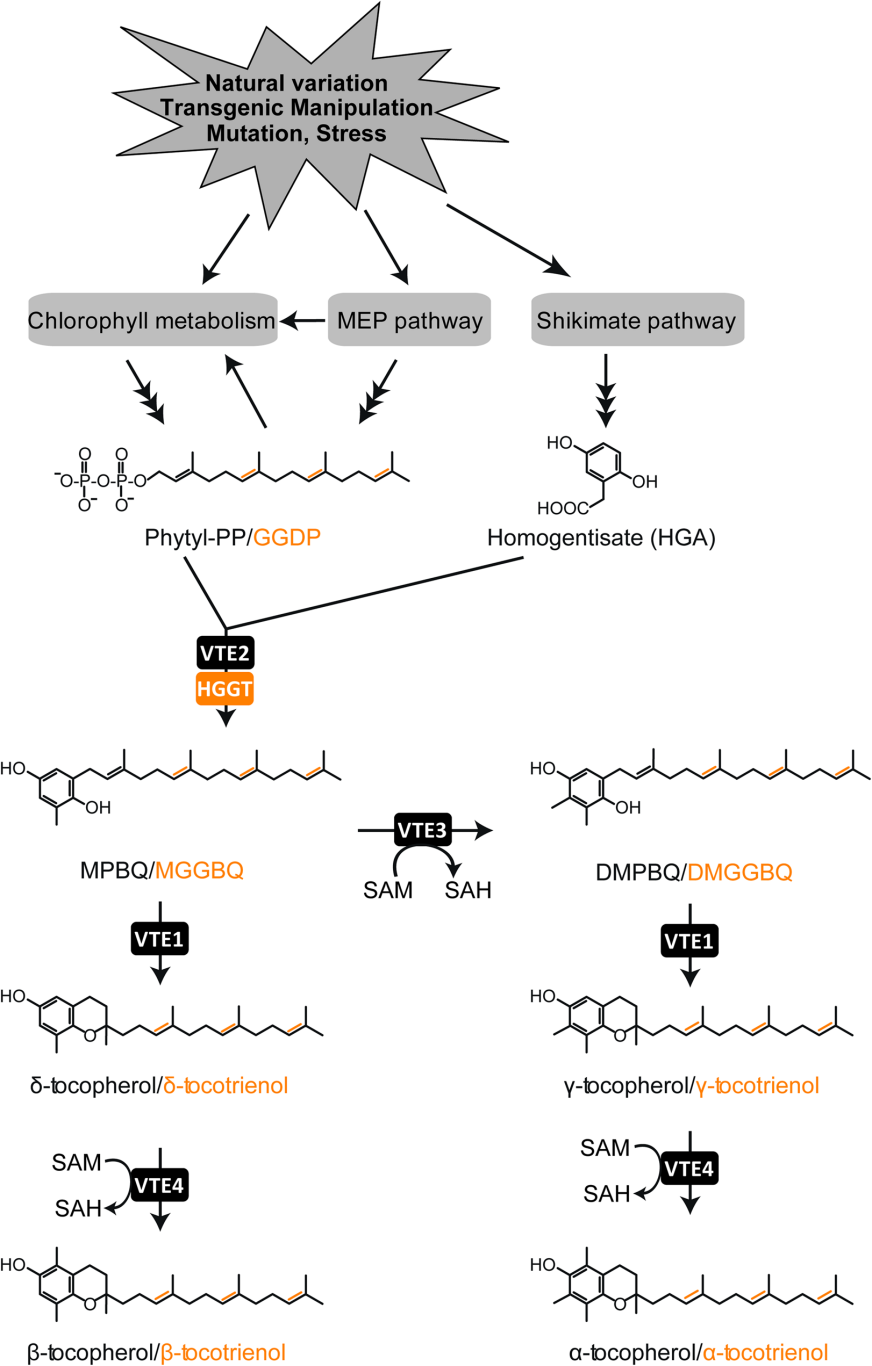
Overview of the tocopherols and tocotrienols synthesis and sources of metabolites. Enzyme abbreviations: VTE2 (homogentisic acid phytyltransferase), HGGT (homogentisate geranylgeranyl transferase), VTE3 (MPBQ/MGGBQ methyltransferase), VTE1 (tocopherol cyclase) and VTE4 (γ-tocopherol methyltransferase). Compound abbreviations: MEP (methylerythritol 4-phosphate), GGDP (geranylgeranyl diphosphate), Phytyl-PP (phytyl diphosphate), MPBQ (2-methyl-6-phytyl-benzoquinol), MGGBQ (2-methyl-6-geranylgeranyl-1,4-benzoquinol), DMPBQ (2,3-dimethyl-5-phytyl-1,4-benzoquinone), DMGGBQ (2,3-dimethyl-5-geranylgeranyl-1,4-benzoquinol), SAM (S-adenosyl-l-methionine), and SAH (S-adenosyl-l-homocysteine)

The VTE2 gene encodes homogentisate phytyltransferase, the committed step in tocopherol synthesis that condenses phytyl-DP (presumed to be derived from chlorophyll) and homogentisic acid (HGA, derived from the shikimate pathway) for generating MPBQ (2-methyl-6-phytyl-1,4-benzoquinol). The committed step to tocotrienol synthesis is a paralog of VTE2 found in monocots, homogentisate geranylgeranyl transferase (HGGT), that preferentially utilizes GGDP and HGA to generate the corresponding geranylgeranylated derivative, MGGBQ (2-methyl-6-geranylgeranyl-1,4-benzoquinol), which is utilized as a substrate by the subsequent pathway enzymes. At this point the pathway branches with MPBQ (or MGGBQ) being used as the substrate for generating DMPBQ (or DMGGBQ) (2,3-dimethyl-5-phytyl-1,4-benzoquinol or 2,3-dimethyl-5-geranylgeranyl-1,4-benzoquinol) by VTE3 (MPBQ/MGGBQ methyltransferase), which is then cyclized by the VTE1 enzyme (tocopherol cyclase) and then methylated by VTE4 (γ-tocopherol methyltransferase) to produce γ- and α-tocopherols (or tocotrienols) respectively. In the alternative branch, VTE1 cyclizes MPBQ to generate δ-tocopherol (or δ-tocotrienol) which is then acted on by VTE4 to produce β-tocopherol or β-tocotrienol [3–6]. A pathway model of tocopherol/tocotrienol biosynthesis is summarized in Fig. 1.

Vegetative plant tissues can dramatically alter tocopherol content and accumulation in response to various stressful conditions [7–12], and we provide a detailed method and examples of using it to assess the impact of high temperature, high light and dark stresses on tocopherol biosynthesis in Arabidopsis leaf tissue (Fig. 1). The same HPLC method used in conjunction with a different extraction method optimized for extraction of mature, dry maize grain and Arabidopsis seed has been used to robustly quantify seed tocochromanols in 1000–5000 member diversity panels of Arabidopsis and maize as well as in T-DNA and Mu-tagged mutant populations to assess gene function [13–15]. These methods should be broadly applicable for similar studies in a wide range of plant species.

## Results and discussion

### Method development with standards and testing with plant tissues

**Fig. 2:**
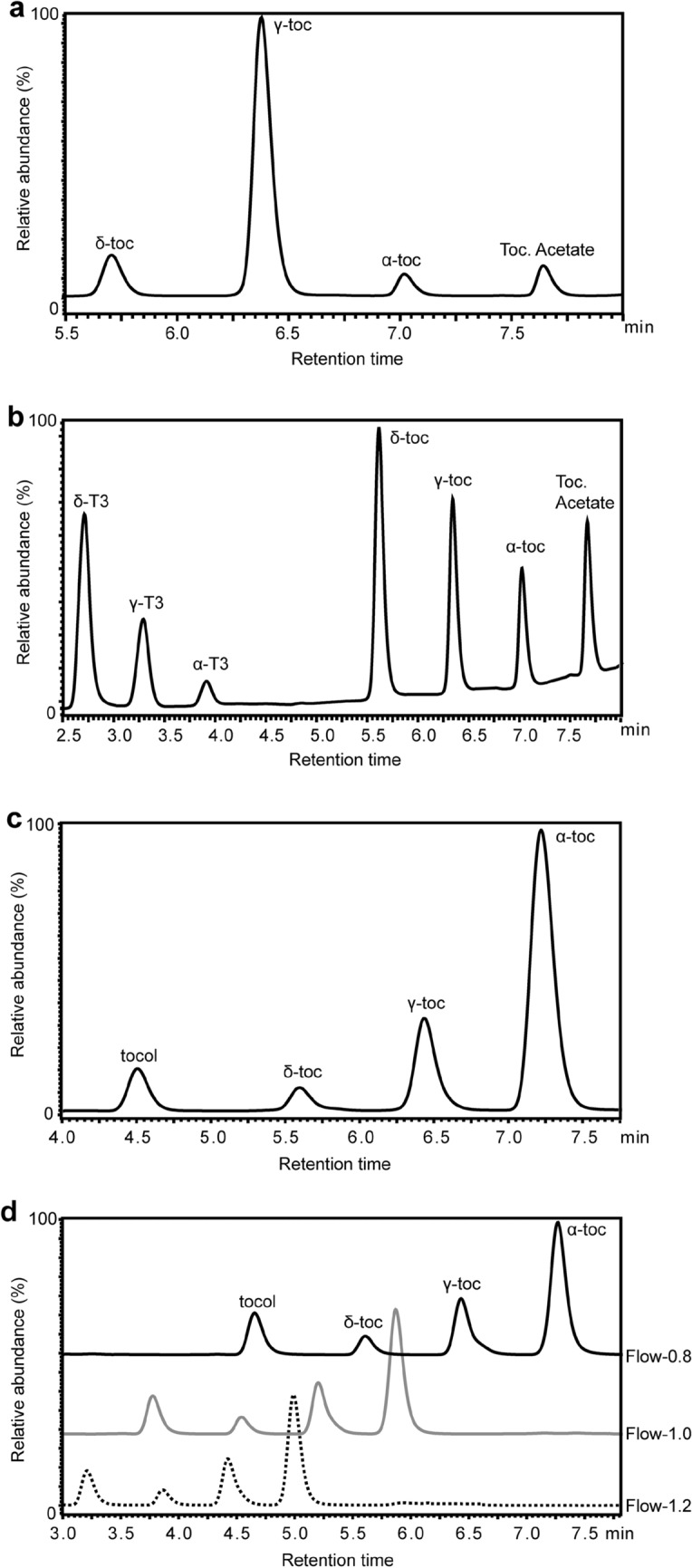
HPLC traces of tocopherol and/or tocotrienol standards. **a** Arabidopsis seed tocopherol standard with tocopherol acetate as an internal standard. **b** maize seed tocopherol and tocotrienol standards with tocopherol acetate as an internal standard. **c** Arabidopsis leaf tocopherol standard with tocol as an internal standard. **d** Arabidopsis leaf tocopherol standards run at three different flow rates: 0.8 ml/min, 1.0 ml/min and 1.2 ml/min

We developed our methods by first separating tocopherol or/and tocotrienol standards by HPLC and then testing the method with various plant extracts. For Arabidopsis and maize seed extraction and separation, we use tocopherol acetate as an internal extraction and recovery control with five serial dilutions of tocopherol standards at levels optimized for the tocochromanol composition of the tissue being analyzed (compare Tables 1 and 2 and Fig. 2a, b). Of several columns and methods tested a Kinetex^®^ 2.6 µm C18 100 Å, LC Column 100 × 4.6 mm with Acetonitrile/Water/TEA and 100% Ethyl Acetate buffers was found to be optimal, giving baseline resolution of tocopherols, tocotrienols and tocopherol acetate with a 13 min cycle time. When this seed method was applied to the analysis of leaf tocochromanols, α-tocopherol levels were found to be highly variable between biological replicates, the cause of which was traced to quenching of α-tocopherol fluorescent signal by overlapping migration of minor chlorophyll species. We assessed other columns and buffers and optimized for both the separation of tocopherols and their resolution from major and minor chlorophyll species. Of several columns and methods tested we found a Waters YMC Carotenoid S-3 3.0 × 100 mm Column with Methanol/Water and 100% *tert*-Butyl methyl ether solvents to be superior for highly reproducible analysis of tocopherols in leaves. For Arabidopsis leaf extraction, we substituted tocol for tocopherol acetate as an internal extraction and recovery control as tocopherol acetate was late in the gradient and subject to quenching as well (Fig. 2c). As with seed analyses, tocopherol standards are run at five serial dilutions at levels optimized for leaf tissue tocochromanol content (Table 3). Below we provide examples of tocopherol analysis in physiologically and genetically diverse leaf samples using our standard 12 min, 0.8 ml/min method. We also show that if needed, by increasing flow rates to 1.0 or 1.2 ml/min the method can be shortened to as little as an 8 min cycle time, with little loss in resolution (Fig. 2d).

**Table 1:** Tocopherol standards used for generating standard curve for Arabidopsis seed

Compound	ng per each 20 μl injection				
	Standard-1	Standard-2	Standard-3	Standard-4	Standard-5
α-Tocopherol	50	25	12.5	6.25	3.125
γ-Tocopherol	500	250	125	62.5	31.25
δ-Tocopherol	50	25	12.5	6.25	3.125

**Table 2:** Tocochromanol standards used for generating standard curve for maize seed

Compound	ng per each 20 μl injection				
	Standard-1	Standard-2	Standard-3	Standard-4	Standard-5
α-Tocotrienol	50	25	12.5	6.25	3.125
γ-Tocotrienol	50	25	12.5	6.25	3.125
δ-Tocotrienol	50	25	12.5	6.25	3.125
α-Tocopherol	50	25	12.5	6.25	3.125
γ-Tocopherol	50	25	12.5	6.25	3.125
δ-Tocopherol	50	25	12.5	6.25	3.125

**Table 3:** Tocopherol standards used for generating leaf analysis standard curve

Compound	ng per each 20 μl injection				
	Standard-1	Standard-2	Standard-3	Standard-4	Standard-5
α-Tocopherol	500	250	125	62.5	31.25
γ-Tocopherol	100	50	25	12.5	6.25
δ-Tocopherol	50	25	12.5	6.25	3.125

### Assessing tocopherol changes under high light stress

**Fig. 3:**
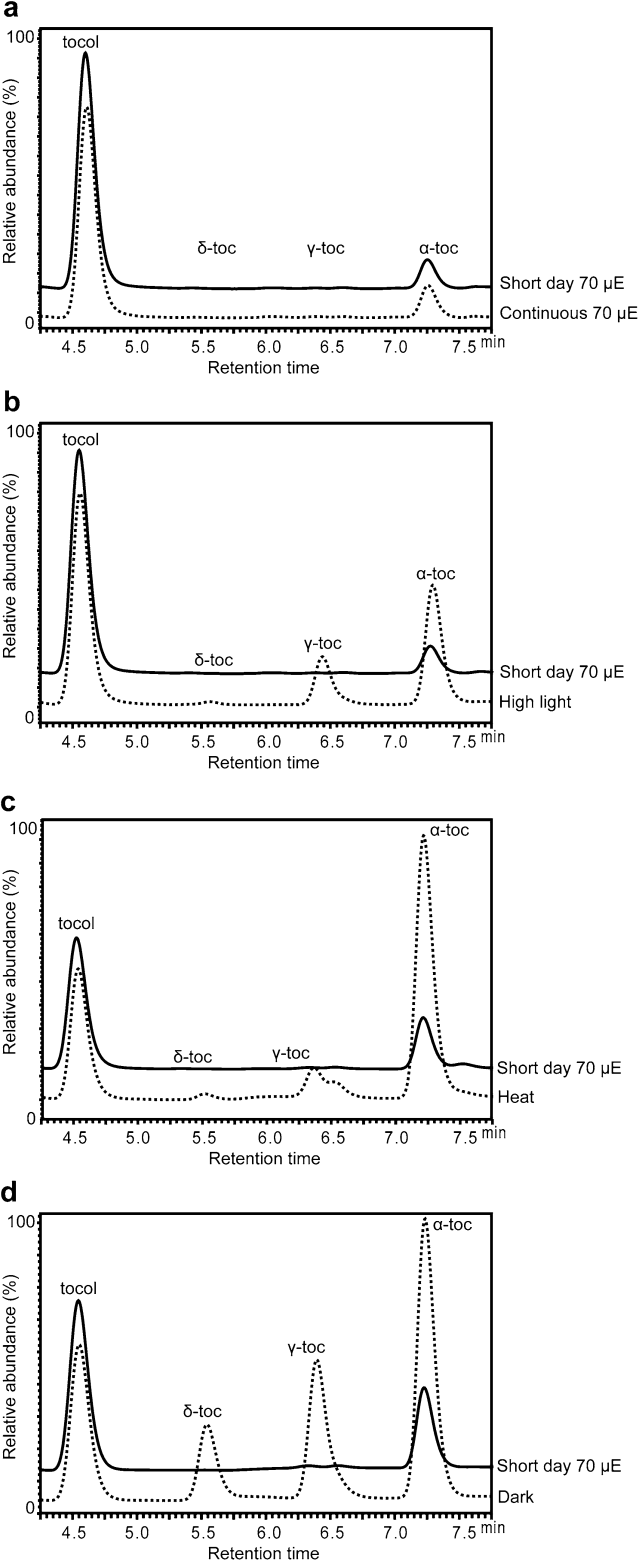
HPLC traces of Arabidopsis leaf tocopherols with and without different stress treatments. HPLC traces of leaf tocopherols after **a** 48 h normal light (~ 70 μE/m^2^/s) and **b** 48 h high light treatment (~ 800 μE/m^2^/s). 4-week-old short-day grown Arabidopsis plants (Control) were switched to continuous normal light and continuous high light, and leaf samples were collected for tocopherol extraction and analysis. **c** HPLC traces of leaf tocopherols after 60 h high temperature treatment. 4-week-old short-day grown Arabidopsis plants were switched from 22 °C to a 37 °C growth chamber with high humidity and continuous light (~ 70 μE/m^2^/s). **d** HPLC traces of leaf tocopherols after 5d dark treatment. Detached leaves from 4-week-old short-day grown Arabidopsis plants were kept on water-soaked filter paper in the dark at 100% humidity. The black trace in each panel is tocopherols present in 4-week-old short-day grown Arabidopsis plants grown at 70 μE/m^2^/s

**Fig. 4:**
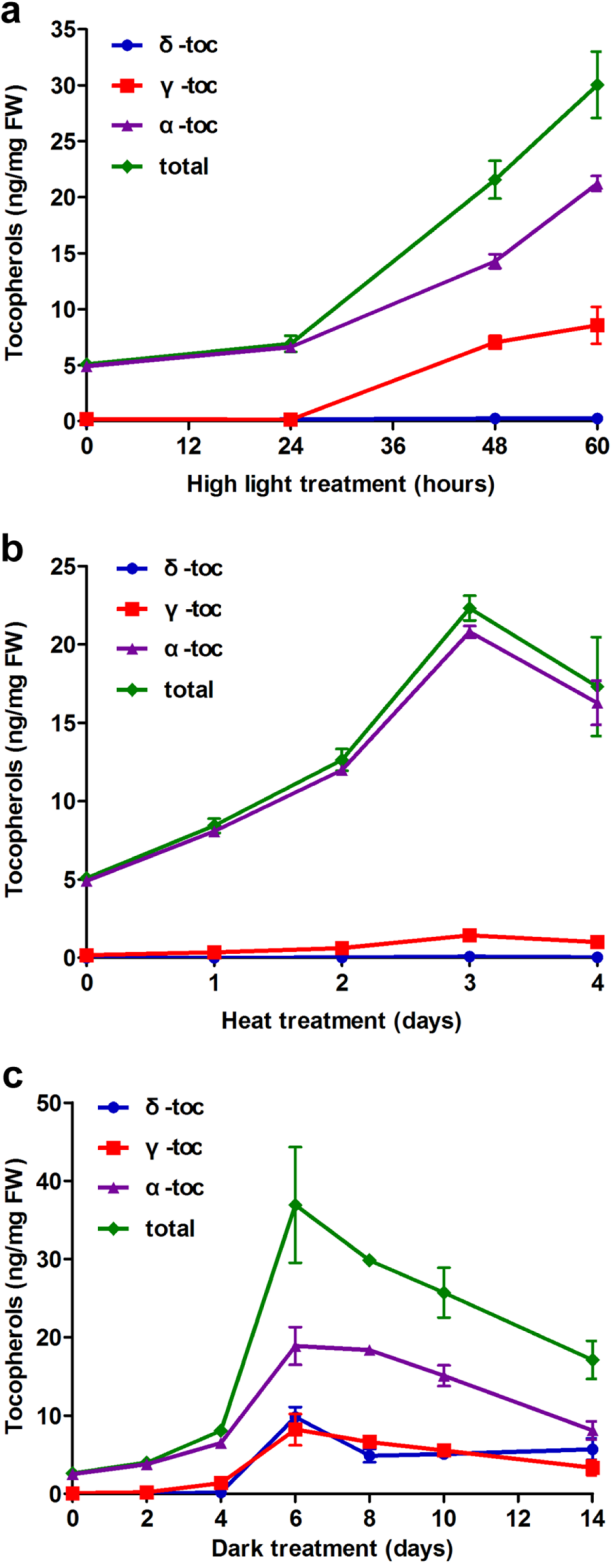
A profile of Arabidopsis leaf tocopherols with different stress treatments. **a** High light treatment impact on tocopherol profiles. 4-week-old short-day grown Arabidopsis plants were exposed to 800 μE/m^2^/s high light and leaf samples were collected every 12 h for tocopherol extraction and run on HPLC. **b** High temperature treatment impact on tocopherol profiles. 4-week-old short-day grown Arabidopsis plants were moved to a 37 °C growth chamber with high humidity and continuous light (~ 800 μE/m^2^/s) for indicated time points. **c** Dark treatment impacts tocopherol profiles. Detached leaves from 4-week-old short-day grown Arabidopsis plants were kept on water-soaked filter paper in the dark at 100% humidity, for the time points indicated. Four biological replicates were sampled and error represents ± SD

High intensity light can cause photooxidative damage to photosystems by creating free radicals [16] and plant responses to this stress including enhance accumulation of tocopherols and other antioxidants [8, 9]. We employed high light intensity (~ 800 μE/m^2^/s) as a stress stimulator for studying changes in tocopherol profiles in a time-dependent manner. 4-week-old Arabidopsis Col-0 plants were grown under short-day conditions and then moved to a growth chamber with continuous 800 μE/m^2^/s light intensity for 12, 24, 48 and 60 h. Without treatment, α-tocopherol accounted for > 95% of total leaf tocopherols, while with 12 h and 24 h treatments, there is a slight increase of α-tocopherol and total tocopherols, but little change in γ-tocopherol. From 24-60 h of high light treatment, there are three- to fivefold increases in α-tocopherol and total tocopherols while γ-tocopherol increased from being barely detectable to a level approximately half that of α-tocopherol at the same time point. For comparison, 48 h of continuous normal light (70 μE/m^2^/s) does not significantly affect total or individual tocopherols (Fig. 3a). It is worth noting that during the 60 h of high light stress treatment, δ-tocopherol levels remain extremely low (Figs. 3b, 4a).

### Assessing tocopherol dynamics during high temperature stress

We next studied the effect of high temperature on tocopherol biosynthesis and composition. High temperature can stimulate a fast turnover of chlorophylls [11] and along with direct reduction of GGDP to phytyl-DP, chlorophyll-derived phytols are thought to be two potential sources of phytl-PP for tocopherol synthesis [1]. Arabidopsis Col-0 plants were grown under short-day conditions for 4 weeks and then moved to a 37 °C high temperature chamber for 1 to 4 days with continuous light (~ 70 μE/m^2^/s). The results indicate that 37 °C high temperature quickly induces α-tocopherol accumulation and overall tocopherol biosynthesis. The highest level of tocopherol accumulation was recorded on the third day of heat stress treatment. From the third to fourth day of treatment, tocopherol amounts dropped from a peak value of 23 ng/mg to 17 ng/mg. γ-tocopherol shows the same trend as α-tocopherol during the 4-day treatment, but it has a peak of only ~ 2 ng/mg on the third day of treatment. Similar to the high light treatment, δ-tocopherol remains very low, near the limits of detection (~ 1 pg/μL), throughout the course of treatment (Figs. 3c, 4b). These results suggest that extended heat stress strongly induces tocopherol biosynthesis, predominantly α-tocopherol.

### Impact of Dark treatment on tocopherols

It has been reported that there is a strong correlation between dark-induced leaf senescence and tocopherol accumulation [8], but a time-dependent understanding of the process is lacking. In a third stress treatment, Arabidopsis Col-0 plants were grown under short-day condition for 4 weeks; then detached leaves were incubated in complete darkness on filter paper soaked with ddH_2_O at 100% humidity [17], and sampled over a 14-day period (Figs. 3d, 4c). Four days of dark treatment approximately doubled the level of total and individual tocopherols (Fig. 4c). By 6 days of dark treatment total tocopherols increased about six-fold to ~ 35 ng/mg, γ-tocopherol five-fold to ~ 6 ng/mg and δ-tocopherol increased from being barely detectable (~ 1 pg/μL) to the second most abundant tocopherol at ~ 9 ng/mg. This strong increase in δ-tocopherol was not observed in either the high light or high temperature treatments. After 6 days, both α- and γ-tocopherol steadily decreased to approximately half their 6-day peak values by 14 days, while δ-tocopherol dropped more slowly to 50% by day 8 and remained constant thereafter at ~ 5 ng/mg (Figs. 3d, 4c).

### Applying the method for profiling leaf tocopherols in Arabidopsis mutants

**Fig. 5:**
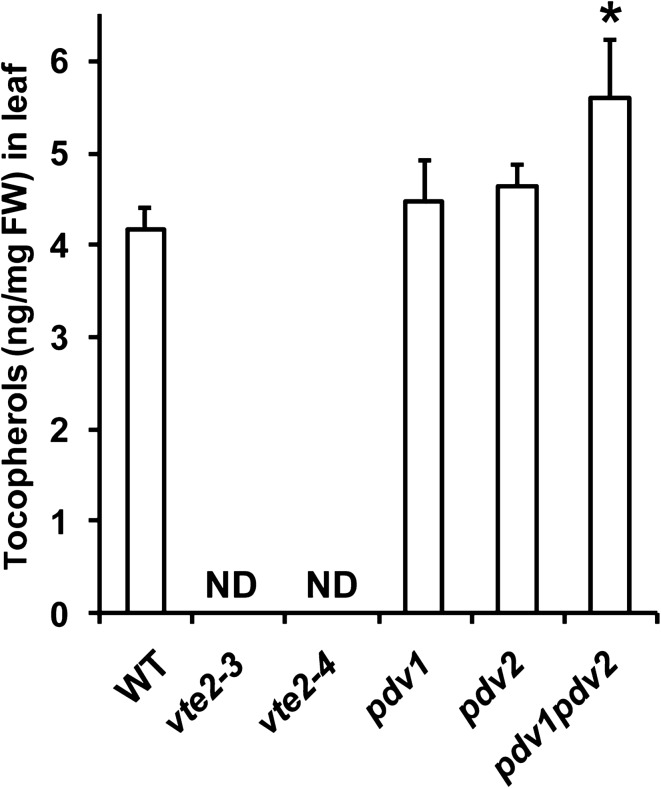
Quantifying leaf tocopherols in various Arabidopsis mutants. Detached leaves were from 4-week-old Col-0 (WT) and mutant plants. *P < 0.05. ND, not detected

To further demonstrate the utility of the method for leaf analysis we applied it to characterizing tocopherols in selected T-DNA mutants of Arabidopsis. We used this method to determine that in two new null alleles for *VTE2* (AT2G18950, *vte2*-*3*, SALK_136065 and *vte2*-*4* CS468581, GK-715D01-02511) that encodes a limiting enzyme in the tocopherol pathway [18], that tocopherol synthesis is completely blocked (Fig. 5). Furthermore, we were able to demonstrate that a more subtle, but still significantly greater amount of tocopherols, was accumulated in the *pdv1 pdv2* double, but not single *pdv* mutant backgrounds (Fig. 5), which severely impacts chloroplast division [19], suggesting a correlation between tocopherol content and chloroplast size/numbers.

### Applying the method for profiling seed tocochromanols in Arabidopsis and maize association panels

**Fig. 6:**
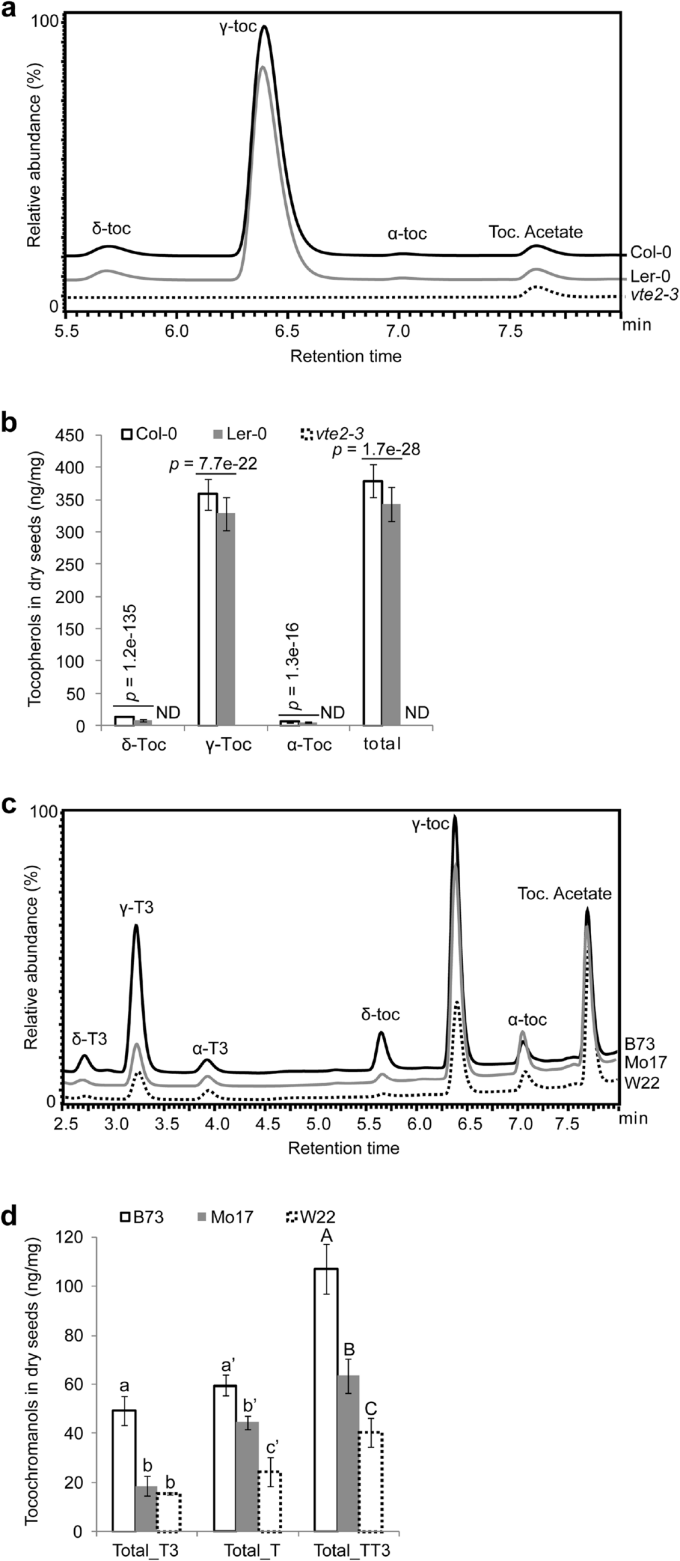
Profiling seed tocochromanols in various Arabidopsis and maize ecotypes. **a** HPLC traces of seed tocopherols in Arabidopsis Col-0, Ler-0 and *vte2*-*3* (Col-0 background) mutant. **b** Quantification of seed tocopherols in Arabidopsis Col-0 (*n* = 154), Ler-0 (*n* = 156) and *vte2*-*3* mutant (*n* = 5); ND, Not-Detected. **c** HPLC traces of seed tocochromanols in maize B73, Mo17 and W22 ecotypes. **d** Quantification of seed tocochromanols in maize B73, Mo17 and W22 ecotypes. Total_T3, total tocotrienols; Total_T, totoal tocopherols; Total_TT3, total tocopherols and total tocotrienols; ND, not-detected

Seeds are the most important plant organ in providing calories and essential nutrients for humanity and seed oils are important dietary sources of vitamin E. Because of the different matrices and composition of dry seed and leaves, a different method for high throughput seed extraction and HPLC separation was developed. This extraction method works equally well for analyzing tocochromanols from Arabidopsis seed or maize kernels and should be applicable to seed of other monocots and dicots. Columbia-0 (Col-0) and Landsberg erecta (Ler-0) are the two most commonly used Arabidopsis ecotypes and Fig. 6a, b show that seed of both ecotypes have similar tocopherol profiles and each tocopherol isoform in Ler-0 that is relatively lower than in Col-0 while the *vte2*-*3* mutant has non-detectable levels of all tocopherols, similar to its phenotype in leaf (Fig. 5). Next, we characterized the seed tocopherols in maize ecotypes B73, Mo17 and W22, which are the three widely used inbreds in maize research. The result indicated that, in addition to tocopherols, maize seeds accumulate tocotrienols as well (Fig. 6c). B73 has ~ 2.5-fold higher tocotrienol level than Mo17 and W22, which are similar, while tocopherol level rankings are B73 > Mo17 > W22. The tocochromanol variation in these three inbred lines suggests substantial natural variation exists for these traits in maize grain. The genetic basis of this variation was recently defined through analysis of tocochromanol traits with this method in the 5000-line U.S. maize nested association mapping panel, which led to the identification of several novel, large effect loci for tocopherols [13]. This study required analysis of two outgrowths of the 5000-member panel which with field controls and standards totaled more than 12,500 extractions and analyses, a testament to the high throughput potential of the methods described in this paper.

## Conclusions and remarks

There are many methods for detecting and quantifying tocochromanols in the literature already; each has its advantages and disadvantages [20, 21]. GCMS (Gas chromatography–mass spectrometry) requires derivatization of samples and ideally inclusion of heavy internal standards added to each sample. Similarly, LCMS (Liquid chromatography–mass spectrometry) methods ideally require heavy standards for accurate quantitation and to compensate for differences in ionization and detection through time, especially when analyzing complex mixtures from divergent ecotypes, inbred lines, physiologically different tissues (e.g., light stressed versus dark-stressed leaves) or fundamentally different matrixes (fresh leaf versus dried seed). When developing the described method for routine extraction and analysis of large numbers (thousands to tens of thousands) of samples from different plant tissues we assessed available methods and settled on the described 96-well extraction procedures for the different tissues due to their ease of use and reproducibility. We selected HPLC fluorescent detection for its combination of robustness and relatively moderate cost of implementation (compared to costs associated with LCMS systems). GCMS and LCMS are certainly superior for more broadly assessing the relative levels of a wide range of compounds, some of which may be tocochromanols or their metabolites [20, 21]. Indeed, and our lab has used GCMS in the past to identify and assess tocopherol recycling metabolites in plants but have found the method to not be amenable or robust for high throughput extraction, derivatization and analysis of crude extracts [9]. While LCMS could be as amenable to high throughput analyses and the described method could be adapted for this approach, the much higher costs of LCMS equipment (or facility charges for use of the machines), maintenance and inclusion of heavy standards for target compounds for accurate quantitation made it prohibitive for the thousands to tens of thousands of samples analyzed.

The extraction and HPLC methods described in this paper were developed to provide a combination of efficient, cost-effective, robust, sensitive and accurate procedures for quantifying tocochromanols in a variety of plant materials and populations and allow for sufficient numbers of timepoints and biological replicates to draw statistically robust conclusions. The limits of detection and limits of quantitation (ng/20 µL injection) are approximately: δ-T = 0.012, γ-T = 0.024 and α-T = 0.12, consistent with a previous study [22]. We monitor recovery using tocol and tocopherol acetate as internal recovery standards, which accelerates the speed of extraction and eliminates the need to do multiple back extractions of samples. Recoveries are consistently around 60% and once an individual sample has been corrected to account for recovery of the tocol or tocopherol acetate internal standards, the method is highly accurate with a reproducibility of  > 92%. A 96 well format plate takes ~ 2 h to extract and if initiated in the morning, HPLC is completed and ready for integration 20 or 22 h later when using the 12-min leaf and 13-min seed gradients, respectively. It is important to note that the same seed or leaf extraction buffers can be used to extract seed or leaf tissues from different plants in parallel and the tocochromanols in the different tissues and plant species can be resolved and quantified with the same HPLC gradient on a single 96 well plate.

We used the leaf method to assess the effects of three divergent stress on the timing of tocopherol accumulation in the Arabidopsis Col-0 ecotype: high light, high temperature and extended dark stress. In all three cases, α-tocopherol and total tocopherols increased dramatically but peaked at different times. The impact of these stresses on δ- and γ-tocopherol isoforms was more varied. For example, γ-tocopherol was only slightly increased during a four-day heat stress treatment but was strongly increased by high light and dark treatments. For δ-tocopherol, there were small changes during the course of high light or high temperature treatments but strong accumulation by dark treatment, which presumably reflects a limitation in γ-tocopherol methyltransferase (VTE4) activity and/or in the level its co-substrate *S*-adenosylmethionine in darkness (Fig. 1). We also assessed the γ/α ratio when the highest amounts of total tocopherol were detected in each treatment. In the case of high light and dark treatments, this ratio is ~ 1:4; while in high temperature treatment, this ratio decreases to ~ 1:10. These results indicate tocopherol isoforms are differentially regulated and accumulated in response to the different stress conditions with α-tocopherol being the most dominant and perhaps most active form for plants in accommodating stressful environments. While stress responses are often studied at the level of mRNA changes, monitoring the dynamic changes of physiologically relevant compounds like tocopherols in a time-resolved fashion can provide important insights into the plasticity of plant response to different environmental stresses.

The described method is ideally suited for quantifying tocochromanols across the large populations of hundreds or thousands of lines needed to assess natural variation in genome wide association studies (GWAS) in leaves or seed of dicots or monocots. Several examples are already published that use this method to phenotype developing kernels of hundreds of sweet corn lines and > 12,500 mature dry grain samples for GWAS of tocochromanols in association panels [13, 15]. While our own work has favored a 12 min sample run time, one can increase the flow rate from 0.8 to 1.2 ml/min (while maintaining the volume of each gradient step) and decrease the total run time to 8 min (Fig. 2d). This flow rate increases backpressure to 3500 to 4000 psi but is well within the range of most standard HPLCs (i.e., non-UHPLCs). An 8-min run time would allow 2 plates to be completed in approximately 26 h on a single instrument. For seed analysis, our 13-min run time allows separation of both tocopherols and tocotrienols. We again find it convenient to use this separation as one can run both Arabidopsis (which lacks tocotrienols) and maize (which has both tocotrienols and tocopherols) on a single plate that is completed in 21 h.

In summary, the described methods provide an efficient, robust and pressure-tested approach to study tocochromanols in both Arabidopsis and maize, which were selected as model dicot and monocots for method development. The study of leaf tocopherol changes with or without various stress treatments adds another tool for assessing plant-environment interactions with a key physiological stress indicator. The high throughput collection of large-scale tocopherol data by HPLC provides ample data for optimized statistical analysis in physiological studies, genome wide association studies and other sampling intensive approaches. This method can also be used for studying tocopherol biosynthesis with or without other different stress treatments in plant species beyond Arabidopsis and maize.

## Methods

### Plant growth conditions and stress treatments

Seeds from different Arabidopsis ecotypes and mutants (all in the Col-0 background) were germinated on ½-MS-0 plates for 5 days and germinated seedlings were transferred to soil and grown under short-day conditions with 8 h-light-22 °C/16 h-dark-18 °C cycles for 4 weeks.

For high light stress treatment, four-week-old Col-0 plants were moved to a high light growth chamber with continuous light, and the first two expanded true leaves were sampled for tocopherol extraction at 12 h, 24 h, 48 h and 60 h time points.

For high temperature treatment, four-week-old Col-0 plants were moved to a 37 °C incubator with continuous light (~ 70 μE/m^2^/s) and the first two expanded leaves were sampled for tocopherol extraction at 1 to 6 day time points.

For dark treatment, the first two expanded leaves from 4-week-old Col-0 plants were detached and kept in darkness on filter paper soaked with ddH_2_O water for 2 to 14 days with samples taken as indicated.

The growth of maize plants was done in the field and harvested as described before [13].

### Isolation of two new *vte2* T-DNA null alleles

Genotyping of *vte2*-*3* (SALK_136065) and *vte2*-*4* (CS468581, GK-715D01-02511) was based on the protocol published by O’Malley et al. [23], using the following primers. *VTE2*-*F*: TGTTGTTGCAGCTCTCATGATG; *VTE2*-*R*: ACCCAGAGTTACAGAGAATGATCG; LB-1.3: ATTTTGCCGATTTCGGAAC (SALK T-DNA border primer), and PAC161-Lb1: CAAGGCATCGATCGTGAAGTTTC (GABI-Kat T-DNA border primer) [24]. Both mutants are in the Col-0 background.

### Leaf tocopherol extraction and HPLC running

Leaf samples from each treatment and time point were quickly frozen with liquid nitrogen, and extractions were performed when all samples were collected. Details for extracting tocopherols from Arabidopsis leaf and seeds are listed below as short protocols following the methods section. HPLC separation and quantification were performed using a Waters YMC Carotenoid S-3 3.0 × 100 mm Column (cat # CT99S031003WT) and the following conditions. 12-min run, 0.8 ml/min gradient (Time, B %): 0–1 min, 100; 1–7.5 min, 76; 7.5–8 min, 0; 8–9.5 min, 0; 9.5–10 min, 100; 10–12 min, 100. 9.6-min run, 1.0 ml/min gradient (Time, B %): 0–0.8 min, 100; 0.8–6 min, 76.3; 6–6.4 min, 0; 6.4–7.6 min, 0; 7.6-8 min, 100; 8–9.6 min, 100. 8-min run, 1.2 ml/min gradient (Time, B %): 0–0.7 min, 100; 0.7–5.1 min, 76; 5.1–5.45 min, 0; 5.45–6.45 min, 0; 6.45–6.78 min, 100, 6.78–8 min, 100. Oven temperature was 30 °C and FLD (Fluorescence detector; Shimadzu model, RF-20A, cat# 228-45147-42) settings were Excitation Wavelength 290 nm and Emission Wavelength 325 nm with attenuation set to medium sensitivity. Solvent B: 90:10 v/v Methanol/Water; Solvent A: 100% MTBE (*tert*-Butyl methyl ether).

### Seed tocopherols extraction and HPLC running

Arabidopsis seeds were harvested and collected in 2 ml Eppendorf tubes, a few pieces of Drierite were added (cat # 238988, Sigma-Aldrich) and the tubes capped and kept at room temperature for at least 4 weeks before weighing and extraction. For corn, ears of field-grown maize were harvested and air-dried at 37 °C for 3 days, hand shelled, ground to powder using IKA tube mill (cat # 0004180001, IKA) under the setting of: speed 25,000 rpm, total time: 2 min, grinding interval time: 5 s with at least 30 s paused between grindings. Details for extracting tocopherols from Arabidopsis and maize seeds are listed as short protocols following the methods section. HPLC separation and quantification were performed using a Kinetex^®^ 2.6 µm C18 100 Å, LC Column 100 × 4.6 mm (cat # 00D-4462-E0, Phenomenex) with the following conditions. Gradient (Time, B %): 0–3 min, 100; 3–5 min, 85; 5–10 min, 30; 10–11.2 min, 0; 11.2–13, 100. Oven temperature was 40 °C and FLD (Fluorescence detector; Shimadzu model, RF-20A, cat# 228-45147-42) settings were Excitation Wavelength 290 nm and Emission Wavelength 330 nm with attenuation set to low sensitivity. Solvent B: 85:15:0.1 v/v/v Acetonitrile/Water/Triethylamine; Solvent A: 100% Ethyl Acetate; Pump flow: 2 ml/min.

### Data processing and statistical analysis

Raw data were collected from HPLC runs using LC Solution (Shimadzu) and processed with Microsoft Excel. Figures for tocopherol profiles were generated by Microsoft Excel and Graphpad Prism.

#### A simplified protocol for tocopherol extraction from leaf tissue

1. Weigh out 25 ~ 50 mg of fresh leaf tissue in a 1.4 ml barcoded MICRONIC tubes (cat # 1775-2607, USA Scientific). We typically use barcoded tubes and plates for tracking samples during processing.
2. Add four 3 mm glass beads to each tube. If not to be used for extraction immediately, seal the tubes with a rubber lid (cat # 1775-3002, USA Scientific), quick-freeze in liquid nitrogen and store at − 80 °C until extraction.
3. If possible, the following steps should be performed in the absence of strong light. Add 450 μl of extraction buffer to each tube, cover the tubes with lid strips (cat # 1775-3002, USA Scientific) and shake for 10 min on a paint shaker (Pacer Industrial Mixers, Pacer Dual 15). Arabidopsis leaf extraction buffer (75 ml): 50 ml Methanol; 25 ml Chloroform; 75 mg BHT (butylated hydroxytoluene); 1.5 ml of 50 ng/μl stock Tocol as an internal standard for recovery calculation (cat # 128-37-0, Sigma).
4. Quick spin on a benchtop centrifuge to remove solvents from lids.
5. Add 300 μl of HPLC grade water to each tube followed by 150 μl of HPLC grade chloroform. Cap the tubes using a new set of lids or strips and vortex for 10 min.
6. Spin for 10 min at 3750 rpm in Thermo Scientific Multifuge X3R centrifuge (cat #: T9FB2185543) at 4 °C.
7. Transfer 200 μl of the organic phase (bottom, green phase) to a new set of evaporation tubes (cat # 1774-2022, USA Scientific) using a multichannel pipette and gel loading tips (cat # 02-707-138, Fisher Scientific).
8. Evaporate the organic phase in a Thermo Scientific Speedvac concentrator (Model: Savant SPD111V) at room temperature. Also evaporate five 450 μl aliquots of extraction buffer containing the internal standard in a 1.5 ml micro centrifuge tube in the same fashion to calculate the 100% recovery rate. Check occasionally to prevent over-drying.
9. Re-suspend extracts by adding 100 μl of methanol, cap the tubes, cover with foil and vortex on Eppendorf MixMate (cat # 5353) for 10 min at 2000 rpm.
10. Spin for 5 min at 3750 rpm at 4 °C. Taking care to not disturb any pellets, transfer supernatant to an HPLC plate using a multichannel pipette. Seal plate with Dot-Scientific seal film (cat # T496) and quick spin to remove any bubbles prior to injection.
11. Transfer calibration standards and internal standards into 1.5 ml vials with 200 μl glass insert, load on HPLC machine with the plate of extracts in the previous step.
12. Inject 20 μl for running on HPLC.

#### A simplified protocol for tocopherols extraction from seed tissue

1. (*Arabidopsis thaliana*, *At*) Weigh out 8 ~ 12 mg of Arabidopsis seed (conditioned by drying for a minimum of 4 weeks post harvest) in 1.4 ml MICRONIC tubes.
  (*Zea mays*, *Zm*) Weigh out 13.5 ~ 16.5 mg of ground dry corn powder in 1.4 ml MICRONIC tubes.
2. Add two 5 mm glass beads to each tube. For corn, if not used for extraction immediately, seal the tubes with a rubber lid, store at − 80 °C until extraction.
3. If possible, the following steps should be performed in the absence of strong light. (*At*) Add 450 μl of Arabidopsis extraction buffer, cover tubes with lid strips and shake for 10 min on a paint shaker. Arabidopsis seed extraction buffer is identical to Arabidopsis leaf extraction buffer except that 1.5 ml of 1 μg/μl stock tocopherol acetate (cat # T3376, Sigma Aldrich) is added (instead of Tocol) as an internal standard for recovery calculation. Note Tocopherol acetate is a different internal standard than that used for Arabidopsis leaf extraction buffer.
  (*Zm*) Add 400 μl of maize extraction buffer followed by 150 μl of HPLC grade chloroform, cover tubes with lid strips and shake for 10 min on a paint shaker. Corn extraction buffer (75 ml): 45 ml Ethyl Acetate; 30 ml Acetone; 75 mg BHT; 1.5 ml of 1 μg/μl stock tocopherol acetate (cat # T3376, Sigma Aldrich) as an internal standard for recovery calculation. Go to step 6 for corn extraction.
4. (*At*) Quick spin on a benchtop centrifuge to remove solvents from lids.
5. (*At*) Add 300 μl of HPLC grade water to each tube followed by 150 μl of HPLC grade chloroform. Cap the tubes using a new set of lids or strips and vortex for 10 min.
6. Spin for 10 min at 3750 rpm in Thermo Scientific Multifuge X3R centrifuge at 4 °C.
7. Transfer 200 μl of the organic phase (bottom, yellow phase (chloroform) for Arabidopsis; upper, yellow phase (acetone) for maize) to a new set of evaporation tubes using a multichannel pipette and gel loading tips.
8. Evaporate the organic phase in a Thermo Scientific Speedvac concentrator at room temperature. Also evaporate five aliquots of extraction buffer containing the internal standard (450 μl for Arabidopsis and 400 μl for mazie) in a 1.5 ml micro centrifuge tube in the same fashion to calculate the 100% recovery rate. Check occasionally to prevent over-drying.
9. Re-suspend extracts by adding 100 μl of fresh 70:30 (v/v) solution of Acetonitrile/Ethyl Acetate, cap the tubes, cover with foil and vortex on Eppendorf MixMate for 10 min at 2000 rpm.
10. Spin for 5 min at 3750 rpm at 4 °C. Taking care to not disturb any pellets, transfer supernatant to an HPLC plate using a multichannel pipette. Seal plate with Dot-Scientific seal film and quick spin to remove any bubbles prior to injection.
11. Transfer calibration standards and internal standards into 1.5 ml vials with 200 μl glass insert, load on HPLC machine with the plate of extracts in the previous step.
12. Inject 20 μl for running on HPLC.

#### Notes

1. If quantification of individual tocopherols or tocotrienols is required, tocopherol standards are purchased from Supelco, USA (cat# a-47783, d-47784, g-47785), tocotrienols standards are purchased from Cayman Chemicals, USA (cat# a-10008377, d-10008513, g-100084940) and dissolved in 100% methanol to make stock solutions at the following concentrations: in seed extraction, 50 ng/μl for all tocopherols and tocotrienols but also make 500 ng/μl stocks for γ-tocopherol; in leaf extraction, 50 ng/μl for δ-tocopherol, 100 ng/μl for γ-tocotrienol and 500 ng/μl for α-tocopherol). Aliquots of the combined tocopherol standard mix are made as follows: to a microcentrifuge tube add 10 μl of each stock solution and store at − 20 °C.
2. For each extraction series, thaw one aliquot of mixed tocopherol standards, and speedvac to dryness just before step #8. Resuspend the dried standards in 900 μl (800μl if for maize seed extraction) of extraction buffer (containing the appropriate Tocol or tocopherol acetate internal recovery standard); take 450 μl (400μl if for maize seed extraction) and mix with 450 μl (400μl if for maize seed extraction) extraction buffer to achieve a twofold dilution.
3. For each seed extraction series, thaw one aliquot of mixed tocopherol standards, and speedvac to dryness just before step #8. Resuspend the dried standards in 900 μl of extraction buffer (containing Tocol for leaf sample extraction, and toco. Acetate for seed sample extraction, for internal recovery standard); take 450 μl and mix with 450 μl extraction buffer to achieve a twofold dilution.
4. Do three more sequential dilutions in the same fashion to have five twofold serial dilutions for generating a standard curve. Dry the five serial dilution standards along with the other samples in step #8. Each 20 μl of calibration standard injected corresponds to the amounts listed in Tables 1, 2 and 3.
5. Process, inject and analyze these standards along with the other tissue samples in steps 9 through 12.
6. The peak areas from each of the five injections is used to construct a standard curve for each tocopherol. Peak areas from each sample after normalizing for recovery using recovery of the internal Tocol or tocopherol acetate standard are used to calculate amounts in each sample using the corresponding standard curve.

## Data Availability

All relevant data can be found in this manuscript.
